# Characterization of a Test for Invasive Breast Cancer Using X-ray Diffraction of Hair—Results of a Clinical Trial

**DOI:** 10.4137/bcbcr.s3596

**Published:** 2009-11-10

**Authors:** Gary L. Corino, Peter W. French, Myungae Lee, Mariam M. Ajaj, Joseph Haklani, Dharmica A.H. Mistry, Kevin Phan, Phillip G. Yuile

**Affiliations:** 1 Fermiscan Pty Ltd, 48 Hunter Street, Sydney, NSW, Australia; 2 Consortium for Advanced Radiation Sources, University of Chicago, Illinois, USA; 3 Radiation Oncology Associates, Mater Hospital, Sydney, NSW, Australia

**Keywords:** X-ray diffraction, hair, breast cancer, randomized clinical trial, synchrotron

## Abstract

**Objective:**

To assess the performance of a test for breast cancer utilizing synchrotron x-ray diffraction analysis of scalp hair from women undergoing diagnostic radiology assessment.

**Design and Setting:**

A double-blinded clinical trial of women who attended diagnostic radiology clinics in Australia.

**Patients:**

1796 women referred for diagnostic radiology, with no previous history of cancer.

**Main Outcome Measures:**

Sensitivity, specificity and accuracy of the hair test analysis compared to the gold standard of imaging followed by biopsy where indicated.

**Results:**

The hair-based assay had an overall accuracy of >77% and a negative predictive value of 99%. For all women, the sensitivity of both mammography and X-ray diffraction alone was 64%, but when used together the sensitivity rose to 86%. The sensitivity of the hair test for women under the age of 70 was 74%.

**Conclusion:**

In this large population trial the association between the presence of breast cancer and an altered hair fibre X-ray diffraction pattern previously reported has been confirmed. It appears that mammography and X-ray diffraction of hair detect different populations of breast cancers, and are synergistic when used together.

## Introduction

There has been a long-term focus on developing methods for early stage detection of cancer in order to maximize treatment outcomes. Breast cancer is one example that clearly demonstrates excellent survival statistics when early-stage disease is treated using current therapies. If all cases of breast cancer could be detected prior to metastasis then there would be a significant reduction of both individual mortality and the economic burden on the community.^1^ Newer diagnostic methods which enhance sensitivity and specificity of current screening modalities are clearly needed to identify women with early stage disease and to supplement the proven role of mammography and breast ultrasound.

There is a recognized association between a person’s overall health and the state of their hair and nails.^2^ Abnormalities in hair and nails can result from alterations in nutrient supply, inflammation, toxins, heavy metals and physical damage. Many of these may be affected by a systemic disease or by a localized malignancy through mechanisms yet to be fully understood.^2^ It is becoming more apparent that hair fibre production is the result of a complex relationship between the follicle and its local environment and systemic factors.^3^ It can therefore be said that the end of the fibre closest to the follicle is most likely to represent the current state of the individual’s health.

The structural arrangement of keratin intermediate filaments in the hair fibre can be determined using X-ray diffraction. Astbury used X-rays to demonstrate that hair contains a crystalline phase,^4^,^5^ and Pauling proposed the alpha-helical secondary structure of hair to account for the resulting X-ray diffraction patterns.^6^

In 1999 James and colleagues reported differences in the small angle X-ray scattering (SAXS) patterns of hair from individuals with breast cancer compared to healthy subjects.^7^ The SAXS patterns of hair from cancer patients contained a ring of comparatively low intensity which was superimposed on the normal α-keratin pattern obtained from healthy control subjects. Subsequent papers from the same group reported SAXS analysis results of blinded human samples which were consistent with the initial publication.^8^,^9^ Pre-clinical data supporting the finding was presented using an animal model of breast cancer.^9^

A study of hairs from cancer and normal subjects using Fourier transform infrared attenuated total reflection provided independent validation of the underlying hypothesis that hair from individuals with breast cancer exhibits structural and compositional abnormalities.^10^

In 2008, the first replication of the finding independent of the original author was reported by two of the present authors.^11^ They reported the results of analysis of hair from 39 women, 15 of whom had confirmed breast cancer. They achieved a sensitivity of 86% and a specificity of 81%. The results were less accurate than reported by James^7^–^9^ but they confirmed an association between the appearance of a ring with a molecular spacing (determined to be 4.76 ± 0.07 nm) and the presence of breast cancer.

Some key questions remain to be resolved. For example, it is unclear from the literature whether any of breast cancer patients were undergoing chemotherapy at the time of hair collection. As chemotherapy alters hair and nail growth this could be an important consideration. To address this issue, this study collected hair from women with no history of cancer of any sort, and prior to a diagnosis of breast cancer being made. A second key question is what is the sensitivity and specificity of the assay in a large population of women presenting for diagnostic screening. This paper reports on the results of a multi-site blinded clinical trial which was undertaken to address these questions.

## Material and Methods

### Study design

The primary aim of the double-blind study was to determine the accuracy (sensitivity and specificity) of using synchrotron-derived X-ray diffraction of hair to detect the presence of invasive breast cancer by reference to the gold standard of imaging (mammography and/or ultrasound) followed by biopsy where indicated in a diagnostic population. Sensitivity was defined as the proportion of all cancers (confirmed by biopsy) that exhibited an X-ray diffraction pattern that contained a circular feature reported to be associated with breast cancer and specificity as the proportion of all patients that were negative either by imaging or by biopsy that gave a normal X-ray diffraction pattern.

### Sample collection and handling

Hair samples were collected from women referred to one of several radiology clinics for a diagnostic examination. This population was chosen rather than a screening population in order to increase the number of cancer cases in the study. Women were recruited to the trial if they were willing and able to provide informed consent and had usable scalp hair at least 30 mm in length. Women were excluded if their scalp hair had been dyed or chemically treated (such as permanent waving) within the previous 4–6 weeks or if they had a history of breast cancer or other cancers (excluding non-melanoma skin cancer and cervical intra-epithelial neoplasia) within 5 years, ensuring that they were not undertaking chemotherapy treatment at time of hair collection. Recruitment was self-selecting – as women arrived at the clinic they were provided with information, and if they were willing to take part and met the criteria, they were recruited, regardless of any other factors.

Scalp hairs (approximately 20) were cut from the region behind the ear, as close to the skin as possible. Recruitment and sample collection were done by a registered nurse who had been trained in Good Clinical Practice. All hair samples were stored in purpose built plastic specimen containers. Samples were assigned a unique identifying number (barcode) at the clinic and then supplied for diffraction analysis with no other identifying information. All patient medical histories were kept on file at the clinic.

X-ray diffraction assessment required the analysis of six individual hair fibres. They were loaded onto specially designed sample holders and transported to the synchrotron by specialist courier (World Courier, Sydney, Australia).

### Synchrotron X-ray diffraction

Synchrotron X-ray diffraction experiments were carried out at the Advanced Photon Source at the Argonne National Laboratory, USA. Analyses were conducted at sector 31ID (SGX-CAT).

The wavelength of X-rays used for the diffraction studies was 1.1168Å and had a resolution (ΔE/E) of 1 × 10^−4^. The sample to detector distance was approximately 1 metre and was under vacuum to reduce background noise due to air-scattered X-rays. System calibration was achieved using the scattering pattern of Silver Behenate.^12^ The beam spot was defined by slits (JJX-ray Denmark) and was 85 μm in the vertical and 300 μm in the horizontal. Each sample was exposed to approximately 1 × 10^14^ photons. The resultant diffraction patterns were collected on a MAR165 CCD detector (www.rayonix.com).

### Image analysis

Hair fibre diffraction patterns were analysed using Saxs15ID^13^ as previously described.^11^

### Statistical analysis

**Figure 1.**
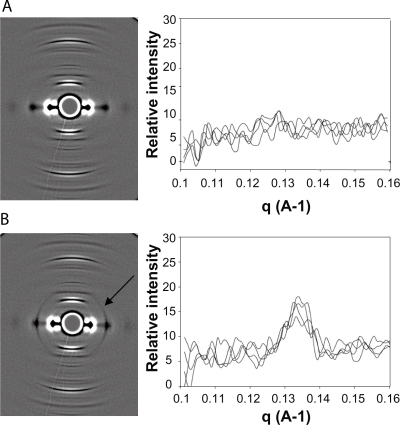
Examples of “positive” (for breast cancer) and “negative” (for breast cancer) hair fibre X-ray diffraction patterns. The raw diffraction data was processed and resulting images were generated using SAXS-15ID as described in the text. **A**) the pattern from a “negative” hair fibre. No circular feature is present, as confirmed by the plot. Note the presence of intense arcs, particularly in the equatorial plane. These are diffraction features regularly observed for *α*-keratin. **B**) the pattern produced from a hair fibre from a woman with breast cancer. Note the circular feature (arrowed) passing through the equatorial spots, at a q space of 1.32 nm^−1^ as defined by the accompanying one-dimensional plot. The other *α*-keratin features are present at similar intensities to those observed in A.

Calls of “positive” or “negative” based on the X-ray diffraction images were made by two analysts blinded to the clinical status of the patient. A negative call was defined as a sample that exhibited the standard alpha-keratin reflections and did not have an additional circular feature superimposed on the pattern. A negative control (a hair fibre from a woman without breast cancer as confirmed by mammography) was used (Fig. 1A). A positive sample was one in which the alpha-keratin reflections were present along with a circular feature^11^ at a q space of 1.32 ± 0.02 nm^−1^ (Fig. 1B). One-dimensional data was extracted from each X-ray diffraction pattern to determine the exact spacing of features in the pattern. The intensity data along a single line starting from the centre of the pattern along the meridional plane at 60°, 120°, 240° and 300°, as previously described.^11^ This process was used to ensure that if a ring was present in the X-ray diffraction pattern, the intensity data would confirm that it was located at the defined q space and was present in all four quadrants. Through analysis of this data, the circular nature of the ring could be established.

The analysts made calls independently and where there was disagreement, they discussed the patterns and made a consensus call. Six hairs were analysed for each subject and a minimum of two hairs displaying the characteristic ring in the zone of interest were required for a sample to be called “positive”. The results of the imaging were re-analysed by two independent radiologists under the supervision of an independent auditor. The pathology results (following biopsy) were also forwarded to the auditor. The diffraction analyses were separately forwarded to the independent auditor, who de-coded the results, matched the imaging and pathology results with the X-ray diffraction data, and communicated the results to the investigators. Sensitivity, specificity, accuracy, positive and negative predictive values and likelihood ratios were calculated using standard definitions.^14^

### Microscopic examination of hairs

To gain a greater understanding of the impact of certain physical characteristics of the hair fibres (diameter, medulla and surface contamination) on the X-ray diffraction patterns, samples were examined by bright field light microscopy.

## Results

### Collection of samples

Scalp hair samples from 1796 women were analysed. The average age of the participants was 52, and 80% were Caucasian (Table 1). There were no adverse events involved in the collection of samples.

### X-ray diffraction patterns

Examples of positive and negative X-ray diffraction patterns are shown at Figure 1. Figure 1A is a representative example of an X-ray diffraction pattern of a hair fibre, from a woman confirmed negative for breast cancer by mammography. The image was processed using SAXS15ID software. The primary alpha-keratin features present along the meridional axis are the 7th order peak (q = 0.94 nm^−1^), the 19th order peak (q = 2.54 nm^−1^) and the 38th order peak (q = 5.08 nm^−1^) of the 46.7 nm lattice. Also seen in the pattern are other features along the meridional axis known to be present in the X-ray diffraction pattern of hair. The region at centre of the diffraction pattern is shielded from the direct beam by a lead beam stop and therefore has not been exposed to X-rays. During image processing this produces an artefactual feature in the centre of the diffraction pattern but has no impact on the region containing the breast cancer ring. The one dimensional plot confirms that there is no peak of intensity present in the image.

In contrast, an X-ray diffraction pattern of a hair from an individual with breast cancer is shown in Figure 1B, along with the one-dimensional plot. Note that all the features seen in Figure 1A are present in Figure 1B with the only difference being the superimposition of a circular feature at a q space of 1.32 nm^−1^ as confirmed by the plot.

### Statistical analysis

#### Overall analysis

The data for the full population is shown at Table 2. Of the 1796 patients, 1775 women in the study had mammographic examination alone or in combination with ultrasound. 1341 had both ultrasound and mammography. 21 of the women had no mammogram result recorded. 197 (11%) were sent for biopsy as a result of a suspicious imaging examination. 39 patients of the 197 that underwent biopsy (19.8%) were confirmed to have invasive breast cancer. The average age of these women was 55 years. 25 of the 39 confirmed breast cancer positives (64%) had a positive X-ray diffraction pattern. Of the 1757 patients with no detectable breast cancer either by biopsy or imaging alone, 394 (22%) had a positive X-ray diffraction pattern. 158 patients (80%) of the 197 who underwent biopsy returned a negative pathology result. Of these, 134 (85%) were negative by X-ray diffraction.

#### Chi-squared test

The observed number of breast cancer positives with a ring was 25, in comparison to an expected number of 9. The difference was significant: χ^2^ (1, N = 1796) = 37.79, p < 0.001.

#### Odds ratio

The OR of breast cancer patients having a positive diffraction test using the data in Table 2 is 6.1775, with 95% CIs 3.5388 to 10.7837.

#### Analysis by age

**Figure 2.**
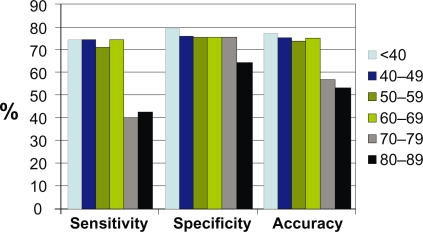
Analysis of the effect of age on the sensitivity, specificity and accuracy of using X-ray diffraction of hair to detect the presence of breast cancer.

Breast cancer patients whose hair X-ray diffraction pattern did not display a ring had an average age of 64.5 years, whilst those with a ring had an average age of 54.8 years. The ability to detect the presence of invasive breast cancer using X-ray diffraction of hair declined significantly for patients over the age of 69 (Fig. 2). When the data were re-analysed excluding women over 69 years of age, there were 27 women with confirmed invasive breast cancer, of whom 20 (74%) were detected using hair X-ray diffraction. The percentage of false positives remained similar to the overall analysis, although for women of any age who were confirmed negative for invasive breast cancer by biopsy, the false positive rate was much lower (24 of 158 patients, 15%).

The calculated statistics for both groups are shown in Table 3. Overall the hair diffraction test had an accuracy of 77%, with a sensitivity of 64% for all women which increased to 74% for women under 70 years of age. It also had a high negative predictive value as demonstrated by the degree of accuracy (85%) with which the test was able to detect a confirmed negative biopsy as negative.

When reviewing the mammographic data in the absence of ultrasound testing, 22 of the 39 cancers gave mammograms that were indicative of the presence of breast cancer, with one or more features such as stellate shape, calcifications, speculations and architectural distortions. One of the cancer patients did not have a mammogram. The other 16 were normal or appeared equivocal. The hair test detected 25 cancers as positive. 33 cancers were positive by either mammography alone or the hair XRD test (Table 4).

#### Microscopic examination of hairs

Hair fibres from false positive samples and true negative samples were retrospectively examined for the presence of medullas and surface contaminants, which have the potential to affect the normal alpha-keratin X-ray diffraction pattern of hair. The false positive samples had significantly more fibres with either medullas or surface particles than the true negative samples. The average diameter of hairs overall was 68 microns. Hair fibres from younger women had only a slightly (10%) larger diameter than that of women over 65 years of age. There was no significant difference in diameter between older and younger fibres in false positives and true negatives (Table 5).

## Discussion

This study was undertaken to compare the results of standard imaging (mammography and/or ultrasound) in detecting cancer in patients undergoing diagnostic imaging with changes in hair structure using X-ray diffraction. The detection rate of cancer by imaging in this series was 39/1796 or 2.17%. This value is consistent with a clinical series self-referring for symptoms and/or self selected for personal high risk, particularly considering the average age which is younger than screening populations (50–69).

The overall specificity of X-ray diffraction analysis of hair to detect breast cancer was 77% for this population. This equates to a false positive detection rate of 22%. A second smaller trial in a similar patient population (213 women) conducted at the same time in a hospital-based clinic showed a similar specificity (78%) (data not shown). The results presented in this study and their statistical analysis support the previously reported correlation between an altered X-ray diffraction pattern of hair and the presence of breast cancer.^7^–^9^,^11^

The data indicate that age is a key factor in determining the accuracy of the test, with sensitivity declining from 80% in women 40 years of age and younger, to around 40% in women over 69 years of age. The reason for this is not known.

Apart from age, there did not appear to be any significant correlation between the usual clinical prognostic markers (tumour size, grade, presence of calcifications, hormone receptors) in those cancers detected by X-ray diffraction and those that X-ray diffraction failed to detect. Two of the patients with breast cancer only had ultrasound examination (no mammography). They were both positive by the hair test. There are several potential mechanisms which could explain the data, and go beyond a simple correlation between a single characteristic of a tumour and the presence of a ring in the X-ray diffraction pattern. One is the possibility of incorporation of extraneous material into the fibre. Increased lipid has been identified in hairs from breast cancer patients.^10^ Another possibility is that secreted cytokines and growth factors from the localised tumour either directly affect the function of hair follicles in laying down keratin intermediate filaments, or affect them indirectly via induction of a host response.

It is important to place this study in the context of the gold standard for screening—mammography. In a 2001 review of historical mammography screening trials,^15^ sensitivity for mammograms alone ranged from 39 to 92%. However, most sensitivity determinations were in the range of 60–66% overall, with Malmo at 61%, Edinburgh 63%, CNBSS-1 (ages 40–49) 61%, and CNBSS-2 (ages 50–59) 66%. These low sensitivity values have been attributed to the fact that most of these trials began many years ago and that mammographic quality and technology has vastly improved. However, A 2007 study in Tochigi, Japan gave a sensitivity for mammography of 61.5%.^16^ Furthermore, using the most advanced technology in the recent Digital Mammographic Imaging Screening Trial (DMIST) coordinated by the American College of Radiology,^17^ the overall sensitivity as defined by a 12-month follow-up period revealed only 70% sensitivity with digital technology and 66% for state-of-the-art film screen technology, comparable to the sensitivities in the other trials. Based on 15-month follow-up, under the assumption that a cancer discovered within a 15-month time frame following a negative mammogram was likely to have been present on the prior study, mammographic sensitivity under this definition was only 41%, and the difference between digital technology and film screen disappeared entirely.

It appears that not all patients with invasive breast cancer exhibit the change in their hair fibres. This is perhaps not surprising. It has been noted that breast cancer is a complex and heterogeneous disease, and that no single model or biological source is expected to mimic all aspects of the disease.^18^ For example, using magnetic resonance spectroscopy, it has been demonstrated that only 80% of breast cancers have a specific membrane signature.^19^ The accuracy of the assay is also affected by variability in the quality and biological characteristics of the hair sample. The presence of medullas may underlie some of the false positives. It is known that various cosmetic hair treatments affect the ultrastructure of hair. This in turn can have a significant impact on the efficacy of the hair diffraction test. In order to collect optimal samples for diffraction analysis, donors should defer such chemical interventions for at least four weeks prior providing their sample. This would allow adequate time for the growth of an untreated segment of hair (approximately 1 cm) and therefore facilitate the diffraction analysis to be conducted on unmodified material.

The estimates of sensitivity and specificity reported here for the hair test are based on the current screening reference standard (imaging with biopsy for positive and equivocal imaging results) and no further testing for the negative imaging results. In so far as the reference standard represents current best practice, the data collected provide information about the extent to which the hair test meets this standard. Its main limitation is in identifying the true sensitivity of the hair test if it was in fact more sensitive than current best practice (fewer false positives). The authors recognise that this may not reflect the ‘true’ accuracy of the hair test in the absence of follow-up data on imaging negatives. A follow-up study on a subset of the patients is being undertaken.

This study however demonstrates that the hair test and mammography detect different but overlapping populations of breast cancers, and thus the hair test may represent a useful adjunct in the diagnosis and even follow up process in the patient with breast cancer but is not a replacement for standard breast imaging at this point. It is hoped that further studies will elucidate the molecular mechanisms which give rise to the ring pattern in breast cancer.

## Figures and Tables

**Table 1.** Ethnicity of trial participants.

**Ethnic group**	**Percentage**
Aboriginal/TSI	0.3
African	0.3
Asian	3.6
Hispanic	0.3
Middle Eastern	1.6
Pacific Islander	0.3
South/Central American	0.3
Caucasian/European	80.1
Not specified	13.3

**Table 2.** Overall results.

	**Patients with breast cancer confirmed by biopsy**	**Patients without breast cancer**		**Total**
		**Confirmed by biopsy**	**Negative by imaging**	
Ring	25 (64%)	24 (15%)	370 (23%)	419 (23%)
No ring	14 (36%)	134 (85%)	1229 (77%)	1377 (77%)
Total	39	158	1599	1796

**Table 3.** Statistical characteristics of the X-ray diffraction test.

**Feature**	**All participants (n = 1796)**	**Participants aged < 70 (n = 1627)**
Sensitivity	64%	74%
Specificity	77.6%	78%
Positive predictive value	6%	5.4%
Negative predictive value	99%	99.4%
Accuracy	77.3%	77.9%
Likelihood ratio of a positive test	2.8	3.3
Likelihood ratio of a negative test	0.05	0.3

**Table 4.** Effect of combining mammography and X-ray diffraction on detection of breast cancer.

	**Cancers detected**	**Sensitivity**
Mammography alone	22	61%
XRD alone	25	64%
Mammograpy and/or XRD	33	85%

**Table 5.** Comparison of microscopic hair characteristics between false positives and true negatives.

	**False pos**	**True neg**	**P value**
Number of fibres analysed	2274	2718	
Percentage of samples with at least one medullated fibre	71.8%	58.1%	
Average no. of medullated fibres per sample	2.1	1.5	<0.0001
Percentage of samples with at least one hair displaying surface contaminants	29.0%	12.4%	
Average no. of fibres with surface contaminants per sample	0.7	0.3	<0.0001
Mean diameter < 55	72	69	0.129
Mean diameter > 65	71	65	0.161
